# High central venous oxygen saturation is associated with mitochondrial dysfunction in septic shock: A prospective observational study

**DOI:** 10.1111/jcmm.15299

**Published:** 2020-04-30

**Authors:** Borwon Wittayachamnankul, Nattayaporn Apaijai, Krongkarn Sutham, Boriboon Chenthanakij, Chalerm Liwsrisakun, Thidarat Jaiwongkam, Siriporn C. Chattipakorn, Nipon Chattipakorn

**Affiliations:** ^1^ Department of Emergency Medicine Faculty of Medicine Chiang Mai University Chiang Mai Thailand; ^2^ Cardiac Electrophysiology Research and Training Center Faculty of Medicine Chiang Mai University Chiang Mai Thailand; ^3^ Center of Excellence in Cardiac Electrophysiology Research Faculty of Medicine Chiang Mai University Chiang Mai Thailand; ^4^ Divison of Pulmonary Critical Care, and Allergy Department of Medicine Faculty of Medicine Chiang Mai University Chiang Mai Thailand; ^5^ Cardiac Electrophysiology Unit Department of Physiology Faculty of Medicine Chiang Mai University Chiang Mai Thailand

**Keywords:** central venous oxygen saturation, mitochondrial function, oxidative stress, Sepsis, serum lactate, venous‐to‐arterial carbon dioxide tension difference

## Abstract

To test the hypothesis that an impaired mitochondrial function is associated with altered central venous oxygen saturation (ScvO_2_), venous‐to‐arterial carbon dioxide tension difference (delta PCO_2_) or serum lactate in sepsis patients. This prospective cohort study was conducted in a single tertiary emergency department between April 2017 and March 2019. Patients with suspected sepsis were included in the study. Serum lactate was obtained in sepsis, ScvO_2_ and delta PCO_2_ were evaluated in septic shock patients. Mitochondrial function was determined from the peripheral blood mononuclear cells. Forty‐six patients with suspected sepsis were included. Of these, twenty patients were septic shock. Mitochondrial oxidative stress levels were increased in the high ScvO_2_ group (ScvO_2_ > 80%, n = 6), compared with the normal (70%‐80%, n = 9) and low ScvO_2_ (<70%, n = 5) groups. A strong linear relationship was observed between the mitochondrial oxidative stress and ScvO_2_ (*r* = .75; *P* = .01). However, mitochondrial respiration was increased in the low ScvO_2_ group. In addition, mitochondrial complex II protein levels were significantly decreased in the high ScvO_2_ group (*P* < .05). Additionally, there was no correlation between serum lactate, delta PCO_2_, and mitochondria oxidative stress or mitochondria function. ScvO_2_ can be potentially useful for developing new therapeutics to reduce mitochondrial dysfunction in septic shock patient.

## 1 INTRODUCTION

Sepsis is the fourth leading cause of death globally, with a mortality rate reaching 70%.^1^ It has been shown that mitochondrial oxidative stress and mitochondrial dysfunction are severe consequences of sepsis.^2^ There is an accumulation of evidence to demonstrate that after resuscitation by standard sepsis care bundle, patients can still potentially die.^3^, ^4^, ^5^ During sepsis, mitochondrial function is compromised. Essential complexes that are compromised are complexes I and IV of the mitochondrial electron transport chain.^6^, ^7^ In addition to inhibition of adenosine triphosphate, the activities of mitochondrial adenosine triphosphate/proton synthase (ATPase/H^+^ synthase) and pyruvate dehydrogenase were inhibited during sepsis,^8^, ^9^, ^10^ causing increased activity of lactate dehydrogenase, which turns pyruvate to lactate.^11^

Currently, none of the physiological parameters used in sepsis care can predict the levels of oxidative stress and mitochondrial dysfunction. It has been proposed that high central venous oxygen saturation (ScvO_2_) reflected low oxygen consumption in tissue level that may be associated with mitochondrial dysfunction or microvascular dysfunction, and increased mortality.^4^, ^12^ However, there are no studies to prove this theory. Lactate level is the standard biological marker to suggest adequate tissue oxygenation. Following resuscitation, if the lactate level is not decreased, this could indicate inadequate resuscitation or an oxygen extraction defect.^13^ Central venous‐to‐arterial carbon dioxide partial pressure (delta PCO_2_) is a physiological parameter, and delta PCO_2_ less than six indicates adequate perfusion.^14^ However, if resuscitation is still not achieved it may indicate mitochondrial dysfunction.^15^, ^16^ Therefore, the ScvO_2_, lactate and delta PCO_2_ levels are potential biomarkers for mitochondrial dysfunction in sepsis. However, the relationship between mitochondrial function and these physiological parameters and clinical score such as Sepsis‐related Organ Failure Assessment (SOFA) in sepsis patients has never been elucidated. Therefore, this study aimed to investigate the relationship between mitochondrial dysfunction in sepsis and ScvO_2_ together with other physiological parameters including serum lactate and delta PCO_2_. We hypothesized that impaired mitochondrial function is associated with increased serum lactate, ScvO_2_ level, delta PCO_2_ and the clinical severity of patients with sepsis.

## 2 MATERIALS AND METHODS

### 2.1 Settings

A prospective study was conducted at Maharaj Nakorn Chiang Mai Hospital, a tertiary hospital in Thailand. This trial was registered at clinicaltrial.gov (NCT03748537), and the protocol was approved by the Institutional Ethical Committee of the Faculty of Medicine, Chiang Mai University (Permit no. EME‐2559‐04262). All adults (age > 18 years old) with suspected sepsis were included in the study, and all participants gave informed consent before their enrolment. Initially, the sepsis was diagnosed by an increase in the Sequential (Sepsis‐related) Organ Failure Assessment (SOFA) score at least two.^17^ The patients with an increase SOFA score of less than two were categorized as infection. The exclusion criteria were pregnancy, patients who became ill from other diseases rather than sepsis, patients who required emergency surgery, documented limitation of therapy order or transferal from another facility. We also recruit eight healthy volunteers matched age and sex with those septic patients for blood analysis.

### 2.2 Measurement

Investigation for source and severity of infection including serum lactate levels were measured in all patients. The whole blood was collected in EDTA tubes from all patients, and the peripheral mononuclear cells (PMBCs) were isolated. Mitochondrial oxidative stress, mitochondrial mass, mitochondrial oxygen consumption‐linked ATP production and mitochondrial oxidative phosphorylation (OXPHOS) protein expressions were determined in the PBMCs,^2^, ^18^, ^19^ and hospital length of stay, 24‐hour mortality rate and 28‐day mortality rate were also documented.

#### 2.2.1 Resuscitation methods

Every septic patient was given empirical antibiotics within one hour after sepsis was suspected. In case of hypotension after isotonic crystalloid fluid 30 mL/kg was load then a vasopressor (norepinephrine being usually the drug of choice) was administered and titrated to achieve MAP ≥ 65 mm Hg, and these for diagnosis septic shock. In patients who diagnosed septic shock and dependent on vasopressor, a central venous catheter, capable of continuous optical haemoglobin ScvO_2_ monitoring (PreSep catheter connected to Vigileo monitor or EV1000, Edwards Lifesciences, UK) was inserted either into a subclavian or internal jugular vein using standard techniques for central venous access. An arterial catheter was used to continuously monitor arterial blood pressure, cardiac output, cardiac index and stroke volume variation (FloTrac sensor, Edwards Lifesciences, UK).

Criteria for adequate fluid resuscitation included any of the following: (a) ultrasonography showing inferior vena cava collapsibility index < 50% during spontaneous breathing or distensibility index < 18% during mechanical ventilation and (b) stroke volume variation < 13% (FloTrac sensor) after the central venous line was inserted. Once the adequate fluid resuscitation criteria were met with either a MAP ≥ 65 mm Hg, the next goal was a ScvO_2_ ≥ 70% (PreSep catheter) or lactate normalization (≤2 mmol/L) within 4 hours of resuscitation. If the ScvO_2_ was < 70% or lactate >2 mmol/L and the post‐fluid resuscitation haemoglobin was <7 g/dL, red cells were then transfused to raise the level of haemoglobin to 7 g/dL or above. If the ScvO_2_ or lactate normalization goal was not achieved after red cell transfusion and the cardiac index was <2.4 L/min/m^2^ (FloTrac sensor), then inotropic support was initiated with dobutamine. Dobutamine initial dosage was 2.5 μg/kg/min given for 30 minutes, and then, it was increased by 2.5 μg/kg/min every thirty minutes until the ScvO_2_ was 70% or greater or lactate normalization. Then, dobutamine was reduced/discontinued at the discretion of the attending clinician. If the ScvO_2_ remained low or lactate >2 mmol/L, then the patient would be intubated, sedated and paralysed, if this had not already been done, to decrease oxygen consumption. Resuscitation was done for the goals of as follows: a mean arterial pressure >65 achieved by fluid resuscitation and vasopressor, and a serum lactate level below 2 mmol/L or a ScvO_2_ > 70%. Therefore, all blood parameters including mitochondrial function measurements as well as ScvO_2_ and delta PCO_2_ were determined after resuscitation when the MAP was greater or equal to 65 mm Hg within 6 hours after arrival at the emergency department.

#### 2.2.2 PBMC isolated and mitochondrial parameter

##### PBMC isolation protocol

Eighteen mL of blood samples was collected from all participants. Once plasma was collected, and PBMCs were isolated using Ficoll density gradient centrifugation. PBMCs were used to measure mitochondrial function, mitochondrial oxidative stress and mitochondrial mass. In brief, the initial centrifugation (1000 g for 10 min) was performed, and the pellet was re‐suspended in phosphate buffer saline solution (PBS). Subsequently, the blood was over‐layered on Ficoll‐Paque reagent (Histopaque, Sigma‐Aldrich) and centrifuged at 400 g for 30 min. After centrifugation, the ring of PBMCs at the Ficoll/plasma interface was collected and then washed twice with 10 mL of PBS. After the last centrifugation, at 1000 g for 10 min, the number of PBMCs was counted using an automatic cell counter (Eve, South Korea). The viability was measured using Trypan blue staining, and 2 × 10^5^ cells of viable PBMCs were used.

##### Mitochondrial oxidative stress determination

To determine mitochondrial oxidative stress levels, the PBMCs (2 × 10^5^ cells) were stained with 5 µmol/L MitoSOX Red (Thermo Fisher) and co‐stained with 100 nmol/L MitoTracker green dye (Thermo Fisher). MitoSOX is a fluorogenic dye that selectively targets the mitochondria. The superoxide oxidizes MitoSOX, and it is excited at 510 nm and emitted at 580 nm. MitoTracker green is a fluorogenic dye that stains the mitochondria regardless of their membrane potential, and it is used to represent the mitochondrial mass. MitoTracker green is excited at 490 nm and emitted at 516 nm. The fluorescent intensity of both MitoSOX and MitoTracker was measured using flow cytometry (FACS Celesta, BD Bioscience). The ratio of fluorescent intensity of MitoSOX and MitoTracker was used to indicate mitochondrial oxidative stress levels in PBMCs from sepsis patients.

##### Mitochondrial function determination

PBMCs (2 × 10^5^ cells) were loaded into an XFe96 culture plate and supplemented with a base medium containing 2 mmol/L of L‐glutamine. Mitochondrial respiration was determined using a mitochondrial stress test kit, and the oxygen consumption rate was measured using a high‐throughput automated 96‐well extracellular flux analyzer (XFe96; Agilent Seahorse). The following reagents were added to determine each mitochondrial function. After basal respiration was measured, 1 µmol/L oligomycin was added to inhibit ATP synthase (complex V), and oxygen consumption‐linked ATP production and proton leak were measured. A 2 µmol/L FCCP (a potent mitochondrial uncoupler) was added as a second compound to determine maximal respiration and spare respiratory capacity. Finally, 0.5 µmol/L rotenone/antimycin A was added to inhibit NADH: ubiquinone oxidoreductase (complex I) and ubiquinol‐cytochrome c reductase (complex III), and non‐mitochondrial respiration were measured. All data were automatically analysed by the Wave software (Wave; Agilent Seahorse).

##### Mitochondrial OXPHOS protein expression determination

The protein was extracted from PBMCs using a radioimmunoprecipitation assay (RIPA) buffer. The total protein (0.3 mg/mL) was mixed with loading buffer, loaded onto 12.5% SDS‐acrylamide gels and then transferred to nitrocellulose membranes in a glycine/methanol‐transfer buffer using a Wet/Tank blotting system (Bio‐Rad Laboratories). Membranes were blocked in 5% non‐fat dry milk in Tris‐Buffered saline and Tween buffer for 1 hour, and the membranes were incubated with anti‐OXPHOS (1:500 dilution; Abcam) overnight at 4°C. Actin (1:1000 dilution; Santa Cruz Biotechnology) was used as a housekeeping protein. Bound antibodies were detected using horseradish peroxidase‐conjugated with antimouse IgG (1:500 dilution; Cell Signaling). The membranes were exposed to an ECL Western blotting substrate (Bio‐Rad Laboratories), and the densitometric analysis was carried out using a ChemiDoc Touch Imaging System (Bio‐Rad Laboratories).

### 2.3 Statistical analysis

Data were presented as median and mean. The statistical analysis was performed using a Student's *t* test and ANOVA test. *P *< .05 was considered statistically significant. The correlation between physiological parameters and mitochondrial function was calculated using Spearman's rho.

## 3 RESULTS

### 3.1 Demographic and clinical characteristics of patients in the sepsis and infectious groups

**FIGURE 1:**
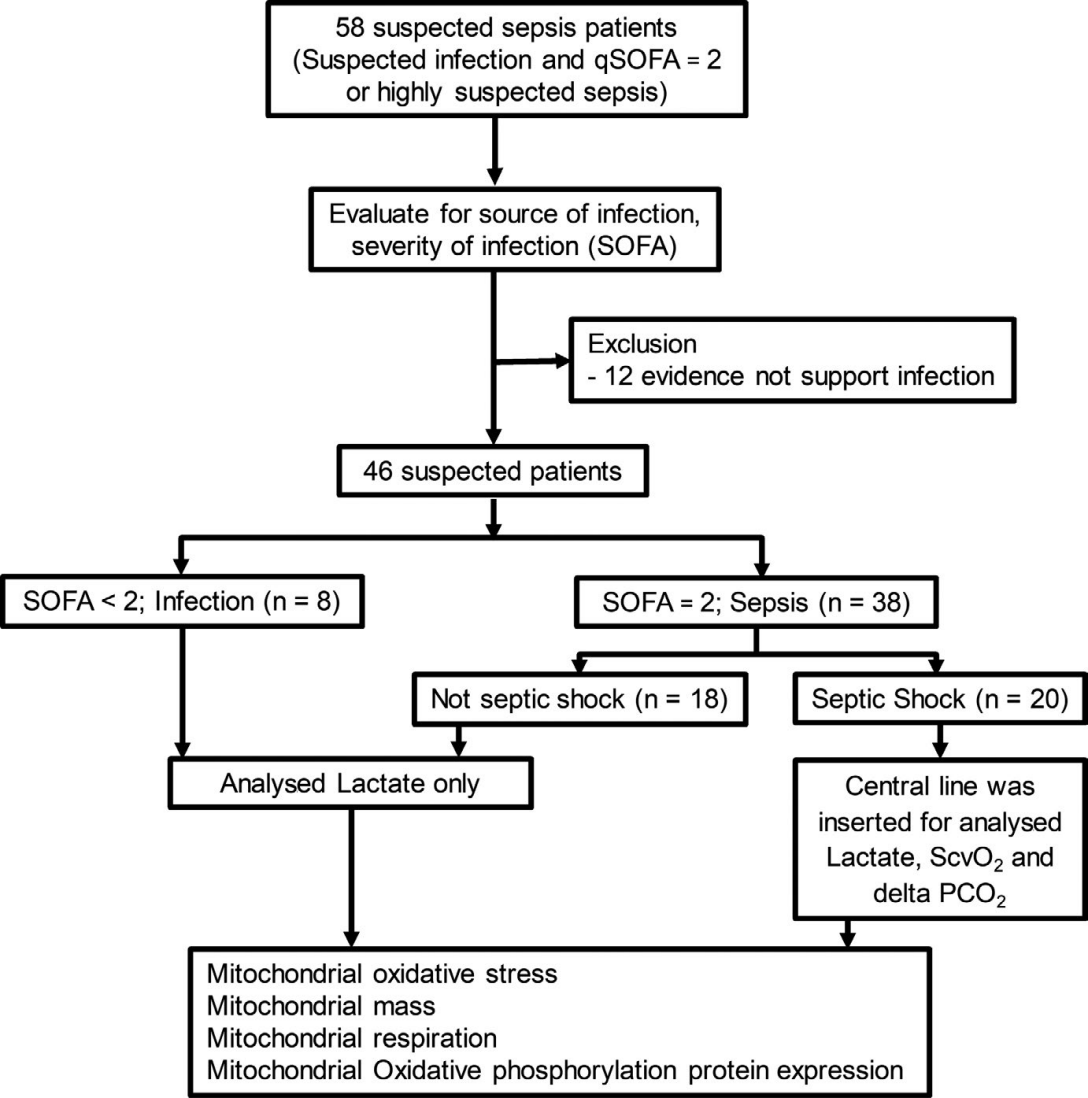
Experimental protocol

Forty‐six patients with suspected sepsis were enrolled in the study. Thirty‐eight patients had increased a SOFA score ≥ 2, so were diagnosed as sepsis, and 8 patients with increased SOFA score < 2 were categorized as the infectious group (Figure 1). Demographic and clinical characteristics including age, sex, the number of patients with medical underlying, source of infection and body temperature were similar between the patients with sepsis and the infectious patients (Table 1). The 24‐hour mortality rate was no different between the sepsis and infectious groups. However, an increased mortality rate was observed 28 days after diagnosis in sepsis patients, compared with infectious patients (23.3% vs 0%, *P* < .05).

**TABLE 1:** Demographic and clinical characteristics of patients with sepsis and those with infection

Parameters	Infection (N = 8)	Sepsis (N = 38)	*P* value
Median age—yr	67 (55‐79)	66.8 (61‐72)	.54
Female sex—no. (%)	3 (37)	16 (42)	.89
Diabetes mellitus—no. (%)	2 (25)	5 (13)	.59
Chronic kidney disease—no. (%)	0	4 (10)	.21
Immunocompromised host—no. (%)	4 (50)	12 (31)	.42
Source of infection—no. (%)			.76
Lung	3 (37)	13 (34)	
KUB	2 (25)	7 (18)	
GI	0	4 (10)	
Skin	1 (12)	3 (7)	
Unknown	2 (25)	11 (28)	
Temperature (°C)	38.4 (37‐39)	38.1 (37‐38)	.54
Median of SOFA score (IQR)	1 (0‐1)	6 (4‐7)	<.01
Median PaO_2_/FiO_2_—mm Hg (IQR)	389 (324‐452)	346 (315‐378)	.31
Median platelet—10^3^ per μL (IQR)	360 (156‐564)	191 (147 ‐ 236)	.02
Median of mean arterial pressure—mm Hg—(95% CI)	74 (64‐85)	70 (62‐78)	.31
Median of total bilirubin level—mg/dL (IQR)	0.64 (0.30‐0.99)	4.06 (0.02‐8.10)	.03
Median of Glasgow Coma Scale score (IQR)	15 (15‐15)	15 (15‐15)	.97
Median creatinine—mg/dL (IQR)	0.9 (0.8‐1)	2.6 (1.2‐4)	.02
Serum lactate—mmol/L	2.3 (1.2‐3.4)	4.8 (3.3‐6.3)	.053
Median of ICU length of stay—d	0 (0‐0)	0 (0‐4)	.72
Median of length of stay—d (IQR)	7 (0‐9)	6 (5‐10)	.70
24‐h mortality—no. (%)	0	4 (10.5)	.20
28‐d mortality—no. (%)	0	9 (23.7)	.049

Abbreviations: FiO_2_, fraction of inspired oxygen; ICU, intensive care unit; IQR, interquartile range; PaO_2_, partial pressure of oxygen; PCO_2_, partial pressure of carbon dioxide; ScvO_2_, central venous oxygen saturation; SOFA, Sepsis‐related Organ Failure Assessment; yr, year.

Sepsis patient were also categorized by lactate level into normal (<2 mmoL/L); borderline (2‐4 mmoL/L) and high (>4 mmoL/L). The clinical characteristics were no differences between groups; however, the SOFA score and 24‐hour mortality were higher in the high lactate group (Table S1). Twenty out of 38 sepsis patients had been diagnosed with septic shock. A central venous catheter was inserted in these patients, and their ScvO_2_ levels and delta PCO_2_ were measured after achieving MAP ≥ 65 mm Hg. The septic shock patients were categorized into 3 groups according to their ScvO_2_ levels: low‐ScvO_2_ (<70%), normal ScvO_2_ (70%‐80%) and high‐ScvO_2_ (>80%). The characteristics of these patients were not different between the groups (Table 2). The 24‐hour mortality rate and 28‐day mortality rate were no differences between groups. There were no differences in the demographic, clinical characteristics and clinical outcome between low (≤6 mm Hg) and high (>6 mm Hg) delta PCO_2_ (Table S2).

**TABLE 2:** Demographic and clinical characteristics of sepsis patients, categorized by central venous oxygen saturation

Parameters	ScvO_2_ (N =20)			*P* value
	<70% (N = 7)	70%‐80% (N = 7)	>80% (N = 6)	
Median age—yr (IQR)	78 (57‐87)	63 (40‐92)	62 (62‐69)	.51
Female sex—no. (%)	3 (43)	3 (43)	2 (33)	.92
Diabetes mellitus—no. (%)	1 (14)	1 (14)	0	.62
Chronic kidney disease—no. (%)	0	1 (14)	0	.04
Immunocompromised host—no. (%)	1 (14)	3 (43)	4 (67)	.16
Median of SOFA score (IQR)	9 (6‐16)	7 (4‐16)	9 (7‐11)	.79
Median PaO_2_/FiO_2_—mm Hg (IQR)	300 (240‐476)	409 (361‐476)	345 (276‐409)	.32
Median platelet—10^3^ per μL (IQR)	27 (19‐184)	176 (91‐296)	144 (40‐193)	.13
Median of mean arterial pressure—mm Hg (95% CI)	62 (49‐72)	62 (54‐69)	58 (52‐62)	.99
Median total bilirubin level—mg/dL (IQR)	1.3 (0.7‐3.5)	1 (0.5‐7.6)	2.1 (0.7‐3.5)	.34
Median of Glasgow Coma Scale score (IQR)	15 (15‐15)	15 (15‐15)	15 (15‐15)	1.00
Median Creatinine—mg/dL (IQR)	1.9 (1.4‐2.5)	2.3 (0.9‐3.9)	2.1 (1.4‐2.7)	.85
Median delta PCO_2_—mm Hg (IQR)	10 (3‐13)	8 (5‐11)	2 (1‐23)	1.00
Median of Serum lactate—mmol/L (IQR)	5.4 (3.7‐16.2)	2.7 (2.5‐22)	4.2 (3.7‐5.4)	.69
Median of ICU length of stay—d (IQR)	1 (0‐52)	0 (0‐0)	0 (0‐0)	.87
Median of length of stay—d (IQR)	8 (1‐15)	11 (5‐52)	6 (4‐20)	.84
24‐h mortality—no. (%)	3 (43)	1 (14)	0	.14
28‐d mortality—no. (%)	3 (43)	2 (29)	1 (16)	.59

Abbreviations: FiO_2_, fraction of inspired oxygen; ICU, intensive care unit; IQR, interquartile range; PaO_2_, partial pressure of oxygen; PCO_2_, partial pressure of carbon dioxide; ScvO_2_, central venous oxygen saturation; SOFA, Sepsis‐related Organ Failure Assessment; yr, year.

### 3.2 Association between mitochondrial oxidative stress and SOFA, ScvO_2_, serum lactate and delta PCO_2_

**FIGURE 2:**
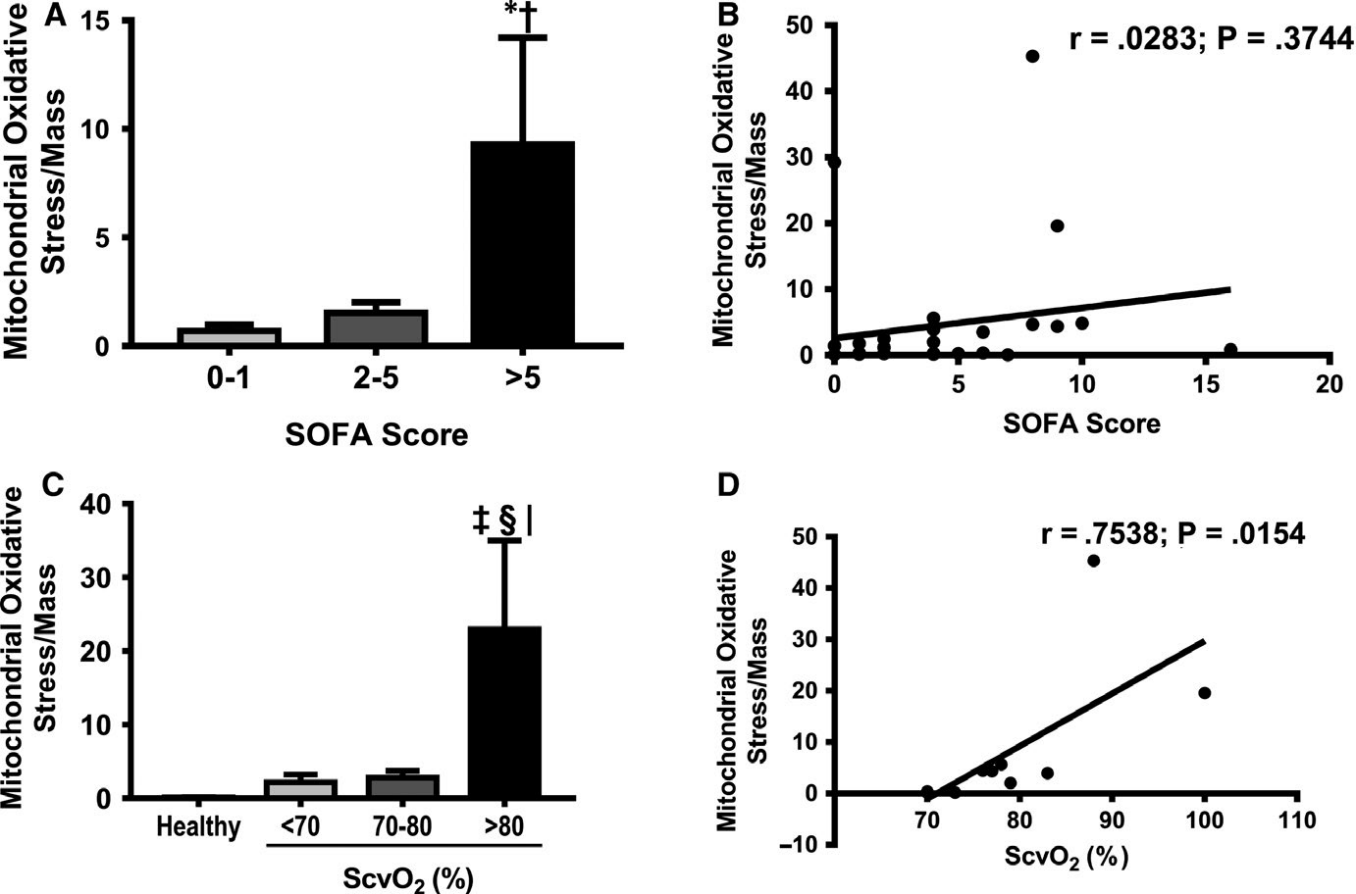
A, The ratio of mitochondrial oxidative stress/mitochondrial mass categorized by SOFA score. B, The correlation analysis between the ratio of mitochondrial oxidative stress/mitochondrial mass and SOFA score. C, The ratio of mitochondrial oxidative stress/mitochondrial mass categorized by ScvO_2_ levels. D, The correlation analysis between the ratio of mitochondrial oxidative stress/mitochondrial mass and ScvO_2_ levels. *P < .05 vs patients with SOFA 0‐1, ^†^ *P* < .05 vs patients with SOFA 2‐5, ^‡^ *P* < .05 vs healthy control, ^§^ *P* < .05 vs septic shock patients with low ScvO_2_, ^|^ *P* < .05 vs septic shock patients with normal ScvO_2._ ATP: adenosine triphosphate; ScvO_2_: central venous oxygen saturation

For SOFA score, all patients were categorized into 3 groups according to the SOFA score, specifically 0‐1, 2‐5 and >5. Our results showed that the ratio of mitochondrial oxidative stress/mitochondrial mass was significantly increased only in patients with a high SOFA score (SOFA score > 5) (Figure 2A). However, the ratio of mitochondrial oxidative stress/mitochondrial mass was not correlated with the SOFA score (Figure 2B). Serum lactate was also not correlated with mitochondrial oxidative stress/mitochondrial mass in sepsis patients.

For ScvO_2_ in septic shock patients, our results showed that mitochondrial oxidative stress and the ratio of mitochondrial oxidative stress/mitochondrial mass were increased only in the high‐ScvO_2_ group, compared to the other groups (Figure 2C). In addition, a strong positive correlation between ScvO_2_ and the ratio of mitochondrial oxidative stress/mitochondrial mass was observed (*r* = .753, *P* < .05) (Figure 2D).

For delta PCO_2_, our results demonstrated that there was no correlation between the ratio of mitochondrial oxidative stress/mitochondrial mass and delta PCO_2_ in septic shock patients.

### 3.3 Association between mitochondrial respiration and SOFA, ScvO_2_, serum lactate and delta PCO_2_

**FIGURE 3:**
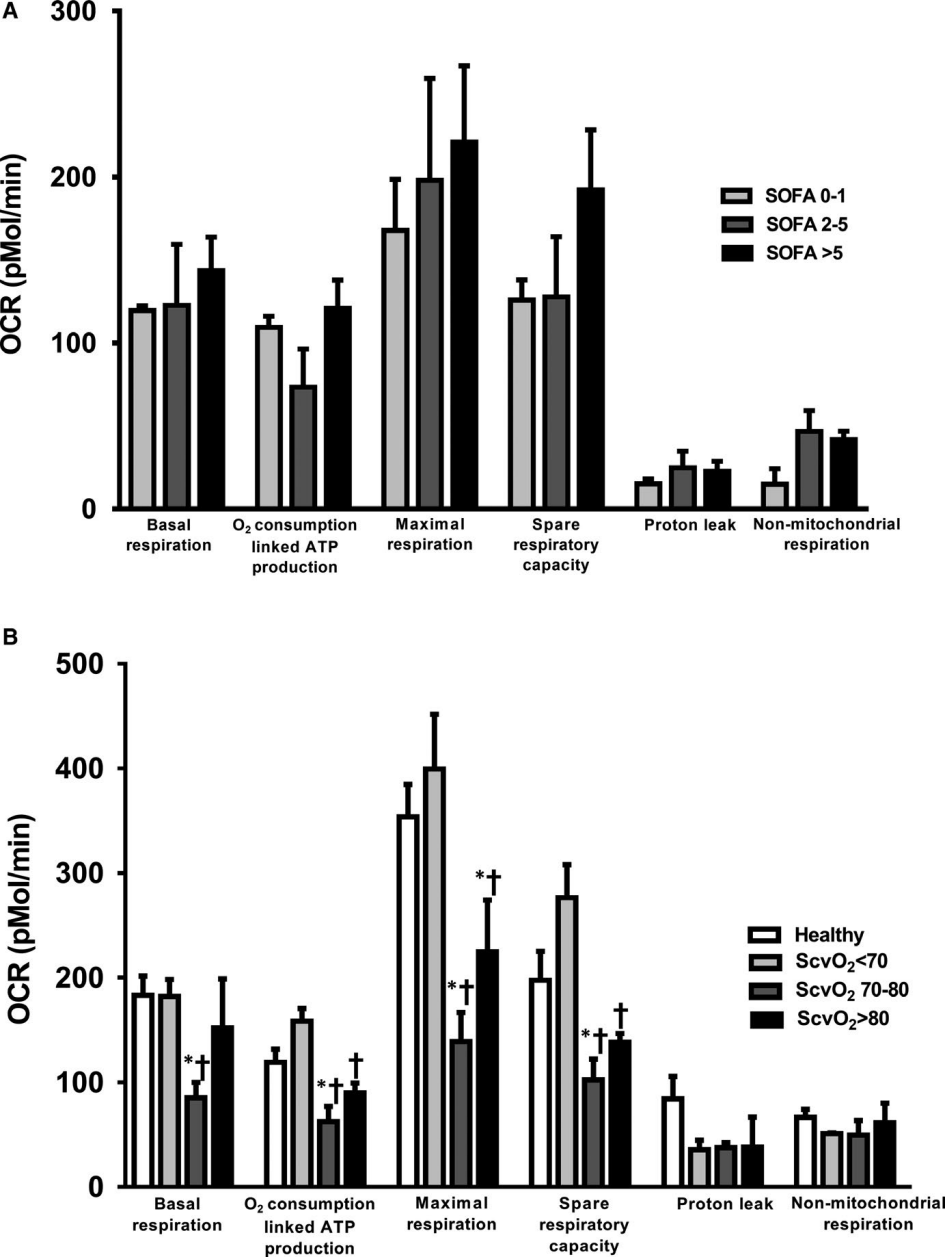
A, Mitochondrial respiration categorized by SOFA score. B, Mitochondrial respiration categorized by ScvO_2_ levels. *P < .05 vs healthy control, ^†^ *P* < .05 vs septic patients with low ScvO_2._ ATP: adenosine triphosphate; OCR: oxygen consumption rate; ScvO_2_: central venous oxygen saturation

For SOFA score, our results demonstrated that all mitochondrial respiration parameters including the basal respiration, oxygen consumption‐linked ATP production, maximal respiration and spare respiratory capacity were not different between SOFA groups in 46 patients (Figure 3A). Serum lactate level was also not correlated with mitochondrial respiration in sepsis patients.

**FIGURE 4:**
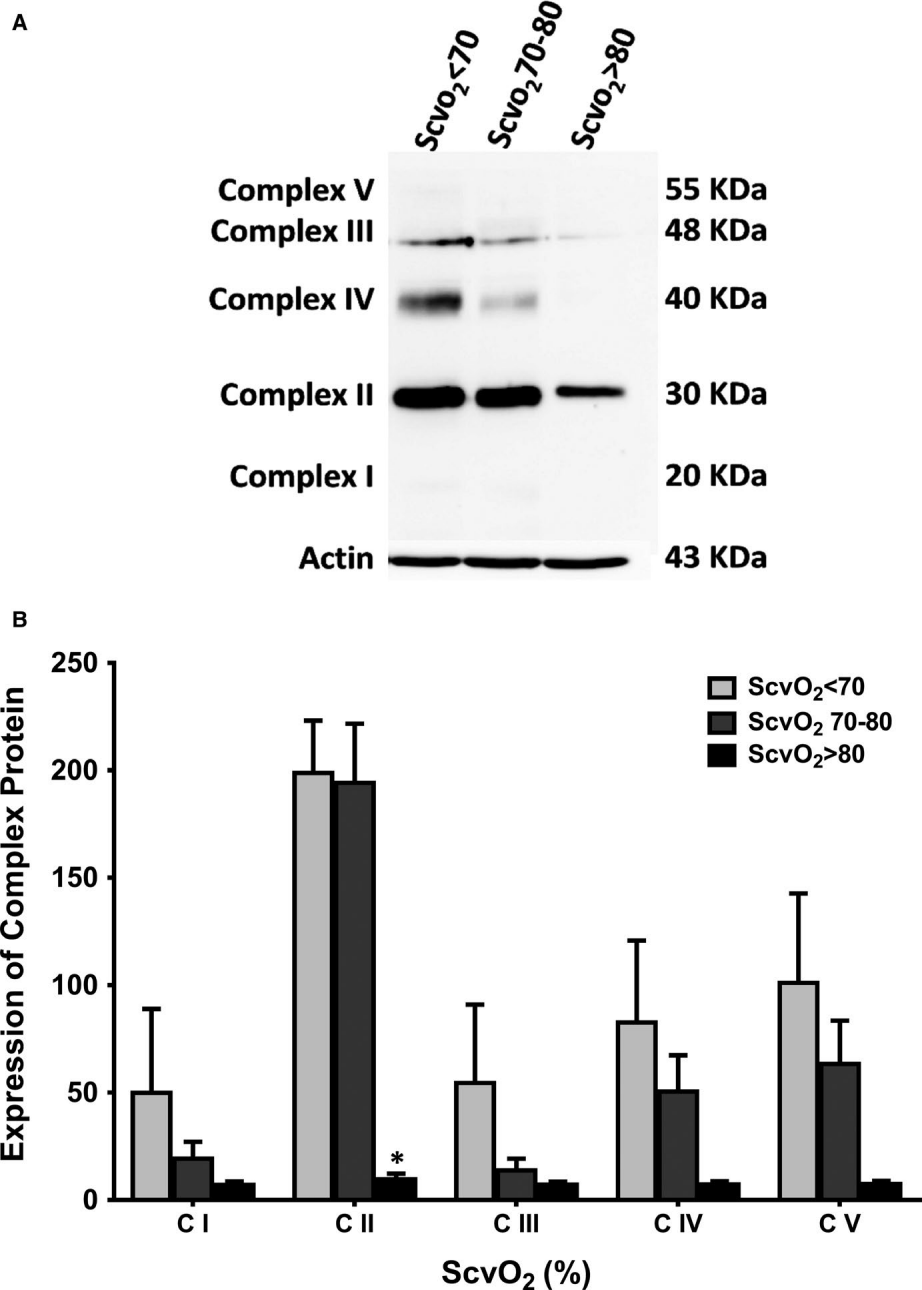
A, Representative image of OXPHOS proteins in PBMCs of patients with sepsis by ScvO_2_ level. B, Mitochondrial oxidative stress phosphorylation protein expression *P < .05 vs normal ScvO_2_ (ScvO_2_ = 70‐80) and low ScvO_2_ (ScvO_2_ < 70). ScvO_2_: central venous oxygen saturation; CI: complex I; CII: complex II; CIII: complex III; CIV: complex IV; CV: complex V

For ScvO_2_ in septic shock patients, data from mitochondrial respiration demonstrated that the basal respiration, oxygen consumption‐linked ATP production, maximal respiration and spare respiratory capacity were similar between healthy and sepsis patients with low ScvO_2_ (Figure 3B). In septic shock patients with normal/high ScvO_2_, oxygen consumption‐linked ATP production, maximal respiration and spare respiratory capacity were lower than healthy and septic shock patients with low ScvO_2_, and there was no statistical difference between septic shock patients with normal and high ScvO_2_ groups. Only septic shock patients with normal ScvO_2_ had a low level of basal respiration when compared to other groups (Figure 3B). In addition, proton leak and non‐mitochondrial respiration were not different among groups. As regards mitochondrial OXPHOS protein expression, our Western blot data showed that the expression of complex II was lower in the high‐ScvO_2_ group compared with the other groups (*P* < .05, Figure 4A,B). For delta PCO_2_, our results demonstrated that there was no correlation between mitochondrial respiration and delta PCO_2_.

## 4 DISCUSSION

Our data demonstrated that mitochondrial oxidative stress was increased only in the high ScvO_2_ group, and mitochondrial respiration was lower in the normal and high ScvO_2_ groups. We aimed to investigate whether high ScvO_2_ was associated with mitochondrial dysfunction.^4^, ^12^ Our data suggest that there was a correlation between mitochondrial oxidative stress and ScvO_2_. A similar trend is also suggested by our findings on the relationship between mitochondrial OXPHOS protein and ScvO_2_. Unexpectedly, this trend was not observed when we investigated the relationship between mitochondrial respiration and ScvO_2_. This is surprising given the biologically close relationship between mitochondrial oxidative stress and mitochondrial respiration. However, the lack of linear correlation between mitochondrial respiration and ScvO_2_ in our septic shock patients could be due to an introduction of a confounding variable including the therapeutic interventions such as volume resuscitation and inotropic support which were received by some sepsis patients who were clinically unstable. Theses therapeutic interventions could have distorted the correlation between predictor variable and outcome variables. Future studies with larger number of patients are needed to verify this hypothesis.

Previous studies demonstrated increased mortality in a high ScvO_2_ group than a normal ScvO_2_ group.^4^, ^12^ High ScvO_2_ could be due to excessive mitochondrial oxidative stress. Mitochondrial dysfunction in sepsis has been associated with mortality.^6^, ^20^ Both human and animal studies also demonstrated that the severity of mitochondrial dysfunction in sepsis was higher than that in cases of hypovolemic and cardiogenic shock,^21^, ^22^ thus indicating the impact of the systemic inflammatory process on mitochondrial dysfunction. A recent study reported that in the early phase of sepsis (Days 1‐4) increased mitochondrial respiratory activity (complex IV; cytochrome oxidase activity per proteins) is associated with improved survival rate.^6^ Moreover, it has been shown that increased late phase (Days 6‐7) activity of mitochondrial respiration (complex I and II) is associated with lower survival rates.^23^ In our study, there was no correlation between mitochondrial oxidative stress/respiration and survival rate. The larger sample size is needed to confirm this finding.

**FIGURE 5:**
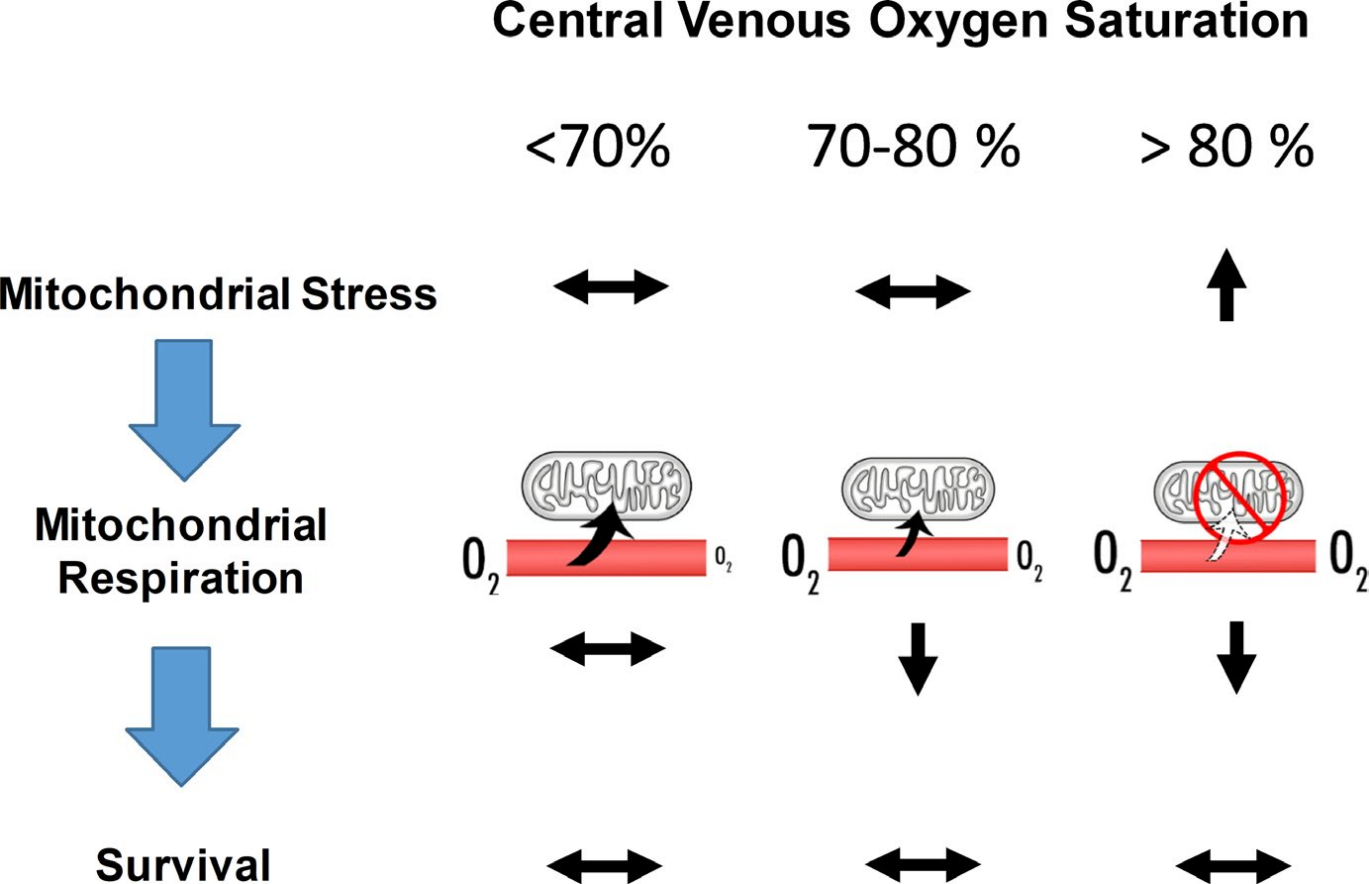
Summary figure of correlation between mitochondrial stress/function and central venous oxygen saturation in sepsis patients referenced to infectious patients. Sepsis patients with high ScvO_2_ had greater mitochondrial oxidative stress than the other groups. Mitochondrial stress began to increase in sepsis patients with ScvO_2_ around 70% and was highest in sepsis patients with high ScvO_2_. This could contribute to poor oxygen extraction in sepsis patients with normal to high ScvO_2_. Double horizontal arrows mean no difference from infection, upwards arrows mean an increase compared than infection and down arrows mean a decrease compared to infection patients

The sepsis surviving guideline mentions that patients with low ScvO_2_ need an aggressive resuscitation.^24^ However, in this group of patients, we found that they had higher mitochondrial respiration than those in the high ScvO_2_ group. This finding suggested that the mitochondria from septic shock patients with low ScvO_2_ group had an average ability to consume oxygen, and this could be due to the levels of OXPHOS protein content was not affected. Sepsis is a dysregulated host response to infection, in which increased pro‐inflammatory cytokines can cause multiple organ dysfunction.^17^ Previous reports showed that inflammatory cytokines directly down‐regulated OXPHOS protein expression.^25^ Moreover, OXPHOS protein expression was decreased in PBMCs from sepsis patients, together with increased inflammatory cytokines levels such as vascular cell adhesion molecule (V‐CAM), intercellular adhesion molecule (I‐CAM) and monocyte chemoattractant protein‐1 (MCP1) in plasma.^2^ Consistent with previous reports, our results showed that OXPHOS protein expression was decreased in sepsis patients. Moreover, our results further demonstrated that mitochondrial oxidative stress was also increased in septic shock patients with high ScvO_2_. This could be due to an elevated electron activity in complex III,^26^ leading to increased oxidative stress. Therefore, a decreased OXPHOS protein expression and increased oxidative stress in our study could lead to a reduction of mitochondrial respiration, and ultimately increased ScvO_2_ in septic shock patients. The summarized diagram showing the correlation between mitochondrial stress/function and central venous oxygen saturation in septic shock patients is shown in Figure 5.

We also found that a 5‐point increase in the SOFA score from the baseline was associated with mitochondrial stress. However, this had no effect on mitochondrial respiration. Since healthy volunteers had been shown to have better mitochondrial function than patients with sepsis,^9^ our results demonstrated that the level of mitochondrial stress was similar between infectious patients without sepsis and patients in the early sepsis phase.

The main limitation of this study is that the number of patients with septic shock was small. In addition, not all patients had the same outcome variable measurements. Of the four major measurements (ScvO_2_, delta PCO_2_, lactate and SOFA), ScvO_2_ and delta PCO_2_ were available only in a subset of patients. This is mainly because insertion of central venous catheter is an invasive procedure; therefore, only severe septic shock patients were justifiable to be inserted (as in 20 septic shock patients in this study). Future studies with larger number of patients are needed to warrant this finding. Moreover, only ROS level was measured in this study. Future study also needs to investigate the role of antioxidant systems in sepsis patients. Since all of the patients in this study received standard treatment of sepsis including antibiotics, norepinephrine and hydrocortisone after resuscitation, future studies are needed to determine the effects of each standard treatment on mitochondrial function in sepsis patients. Finally, there is no standard cut‐off level of mitochondrial dysfunction available at this time in septic patients. Future studies are needed to focus more on the possible interventions into mitochondrial dysfunction in septic patients, especially in those with a high ScvO_2_. Since our findings suggest that reducing ScvO_2_ after treatment may indicate improvement of mitochondrial function, the mitochondrial dysfunction state should be further investigated using the measure of lactate normalization along with ScvO_2_.

## 5 CONCLUSION

This is the first demonstration that ScvO_2_ could be used as a potential marker for mitochondrial dysfunction in sepsis. Moreover, high ScvO_2_ may indicate the need for further intervention on mitochondrial respiration to attenuate mitochondrial dysfunction in septic patients.

## CONFLICT OF INTEREST

The authors declare that there is no conflict of interest.

## AUTHOR CONTRIBUTIONS

BW and NA contributed to data collection, data analyses and manuscript writing. KS, BC, CL and TJ contributed to data collection and data analyses. NC, SCC contributed to study design, data analyses, manuscript editing and final approval of manuscript.

## ETHICS APPROVAL AND CONSENT TO PARTICIPATE

This study was approved by the Institutional Ethical Committee of the Faculty of Medicine, Chiang Mai University (Permit no. EME‐2559‐04262).

## Supporting information

Table S1‐S2

## Data Availability

The data that support the findings of this study are already provided in this manuscript.
