# Supplementary material for: High central venous oxygen saturation is associated with mitochondrial dysfunction in septic shock: A prospective observational study
Source: J Cell Mol Med. 2020 Apr 30;24(11):6485–94. doi: 10.1111/jcmm.15299 (PMC7294163; doi:10.1111/jcmm.15299)
Supplement: Supplementary file 1 — Table S1‐S2 [file JCMM-24-6485-s001.docx]

**Supplementary table 1 Demographic and clinical characteristics of sepsis and infection patients, categorized by the lactate level**

| **Parameters** | **lactate (N=46)** | | |  |
| --- | --- | --- | --- | --- |
|  | **< 2 mmoL/L (N=13)** | **2-4 mmol/L (N=19)** | **> 4 mmol/L**  **(N=14)** | **P value** |
| Median age - yr (IQR) | 78 (57-87) | 63 (40-92) | 62 (62-69) | 0.20 |
| Female sex - no. (%) | 7 (54) | 10 (50) | 2 (15) | 0.08 |
| Diabetes mellitus - no. (%) | 2 (15) | 3 (15) | 2 (15) | 0.36 |
| Chronic kidney disease - no. (%) | 2 (15) | 2 (10) | 0 | 1.00 |
| Immunocompromised host – no. (%) | 8 (62) | 4 (20) | 4 (31) | 0.37 |
| Median of SOFA score (IQR) | 9 (6-16) | 7 (4-16) | 9 (7-11) | 0.04 |
| Median PaO_2_/FiO_2_ – mmHg (IQR) | 362 (286-433) | 362 (262-433) | 421 (300-433) | 0.46 |
| Median platelet – 10^3^ per μL (IQR) | 244 (159-352) | 193 (142-275) | 17.7 (27-195) | 0.15 |
| Median of mean arterial pressure -mmHg (95%CI) | 71 (69-99) | 69 (61-86) | 61 (50-72) | 0.65 |
| Median total bilirubin level - mg/dL (IQR) | 0.4 (0.4-0.6) | 1.2 (0.5-1.9) | 1.5 (0.8-3.5) | 0.02 |
| Median of Glasgow Coma Scale score (IQR) | 15 (15-15) | 15 (15-15) | 15 (14-15) | 0.11 |
| Median creatinine - mg/dL (IQR) | 0.9 (0.8-1.4) | 1.6 (0.9-2.7) | 1.8 (1.2-2.4) | 0.048 |
| Median ScvO_2_ - % (IQR) | 74 (74-74) | 77 (73-83) | 75 (43-83) | 0.87 |
| Median delta PCO_2_ - mmHg (IQR) | 11 (11-11) | 7 (5-8) | 12 (3-19) | 0.56 |
| Median of ICU length of stay – day (IQR) | 0 (0-0) | 1 (0-0) | 0 (0-1) | 0.42 |
| Median of length of stay - day (IQR) | 4 (2-7) | 9 (6-51) | 6 (1-10) | < 0.01 |
| 24 hour-mortality - no. (%) | 0 | 0 | 4 (29) | < 0.01 |
| 28 day-mortality - no. (%) | 2 (15) | 2 (11) | 5 (36) | 0.12 |

FiO_2_, Fraction of inspired oxygen; ICU, Intensive care unit; IQR, Interquartile range; PaO_2_, Partial pressure of oxygen; PCO_2_, Partial pressure of carbon dioxide; ScvO_2_, central venous oxygen saturation; SOFA, Sepsis-related Organ Failure Assessment; yr, year

**Supplementary table 2 Demographic and clinical characteristics of sepsis patients, categorized by central venous-to-arterial carbon dioxide partial pressure difference**

| Parameters | Delta PCO_2_ (N=20) | | P value |
| --- | --- | --- | --- |
|  | **≤ 6 mmHg**  **(N=7)** | **> 6 mmHg**  **(N=13)** |  |
| Median age - yr (IQR) | 69 (62-80) | 62 (54-67) | 0.24 |
| Female sex - no. (%) | 2 (29) | 6 (46) | 0.64 |
| Diabetes mellitus - no. (%) | 1 (14) | 1 (8) | 1.00 |
| Chronic kidney disease - no. (%) | 0.00 | 1 (8) | 1.00 |
| Immunocompromised host – no. (%) | 3 (43) | 5 (38) | 1.00 |
| Median of SOFA score (IQR) | 6 (4-8) | 9 (4-10) | 0.49 |
| Median PaO_2_/FiO_2_ - mmHg (IQR) | 286 (228-438) | 400 (310-433) | 0.44 |
| Median Platelet - 10^3^ per μL (IQR) | 183 (128-296) | 91 (37-154) | 0.14 |
| Median Mean arterial pressure -mmHg (95%CI) | 64 (54-72) | 60 (50-62) | 0.16 |
| Median total bilirubin level - mg/dL (IQR) | 0.8 (0.7-3.5) | 1.6 (1.0-3.5) | 0.70 |
| Median of Glasgow Coma Scale score (IQR) | 15 (15-15) | 15 (15-15) | 0.44 |
| Median Creatinine - mg/dL (IQR) | 1.5 (1.1-2.5) | 2.4 (1.1-3.6) | 0.48 |
| Median of Serum lactate – mmol/L (IQR) | 4.4 (2.8-13.1) | 3.7 (2.7-7.0) | 0.49 |
| Median ScvO_2_ - % (IQR) | 79 (70-83) | 74 (43-79) | 0.39 |
| Median of ICU length of stay – day (IQR) | 0 (0-0)) | 0 (0-4) | 0.64 |
| Median of length of stay - day (IQR) | 5 (3-25) | 6 (1-15) | 0.82 |
| 24 hour-mortality - no. (%) | 0.00 | 4 (31) | 0.26 |
| 28 day-mortality - no. (%) | 1 (14) | 5 (39) | 0.49 |

FiO_2_, Fraction of inspired oxygen; ICU, Intensive care unit; IQR, Interquartile range; PaO_2_, Partial pressure of oxygen; PCO_2_, Partial pressure of carbon dioxide; ScvO_2_, central venous oxygen saturation; SOFA, Sepsis-related Organ Failure Assessment; yr, year
